# The United Kingdom Childhood Cancer Study of exposure to domestic sources of ionising radiation: 1: radon gas

**DOI:** 10.1038/sj.bjc.6600276

**Published:** 2002-06-05

**Authors:** UK Childhood Cancer Study Investigators

**Affiliations:** UKCCS, University of Leeds, Institute of Epidemiology, 30 Hyde Terrace, Leeds LS2 9LN, UK

**Keywords:** radon, childhood cancer risks, leukaemia, case–control

## Abstract

This paper reports the results of the United Kingdom Childhood Cancer Study relating to risks associated with radon concentrations in participants homes at the time of diagnosis of cancer and for at least 6 months before. Results are given for 2226 case and 3773 control homes. No evidence to support an association between higher radon concentrations and risk of any of the childhood cancers was found. Indeed, evidence of decreasing cancer risks with increasing radon concentrations was observed. Adjustment for deprivation score for area of residence made little difference to this trend and similar patterns were evident in all regions and in all diagnostic groups. The study suggests that control houses had more features, such as double glazing and central heating, leading to higher radon levels than case houses. Further, case houses have features more likely to lead to lower radon levels, e.g. living-rooms above ground level. Consequently the case–control differences could have arisen because of differences between houses associated with deprivation that are not adequately allowed for by the deprivation score.

*British Journal of Cancer* (2002) **86**, 1721–1726. doi:10.1038/sj.bjc.6600276 www.bjcancer.com

© 2002 Cancer Research UK

## 

The UK Childhood Cancer Study (UKCCS) was specifically designed to investigate the impact of a wide range of possible risk factors, including exposure to naturally occurring ionising radiation (UKCCS Investigators, 2000). The hypothesis was created partly because of concerns expressed about the possibility that domestic levels of radon gas and its decay products might prove to be a risk for the development of leukaemia in both adults and children. Two distinct lines of investigation had led to these concerns. Firstly, some epidemiological correlation studies appeared to show associations between mean concentrations of radon and leukaemia at all ages (Lucie, 1989), in childhood ALL (Alexander, 1990) and in certain childhood malignancies, including leukaemia (Henshaw *et al*, 1990), although one childhood case–control study failed to confirm these observations (Stjernfeldt *et al*, 1987). Secondly, there were some dosimetric concerns that the bone marrow dose from domestic exposure to radon and its short-lived decay products might be higher than originally thought due to internal patterns of fat deposition (Richardson *et al*, 1991). In addition, risk estimates, based on standard radiological protection approaches, have, more recently, suggested that about 14% of leukaemia in those aged 0–24 in the UK could be due to natural high linear energy transfer (LET) radiation including radon (Committee on Medical Aspects of Radiation in the Environment, 1996; Simmonds *et al*, 1995).

The present report compares the radon concentrations of homes that children were living in at the time their cancer was diagnosed, with those of a similar group of children who did not have cancer.

## MATERIALS AND METHODS

**Figure 1:**
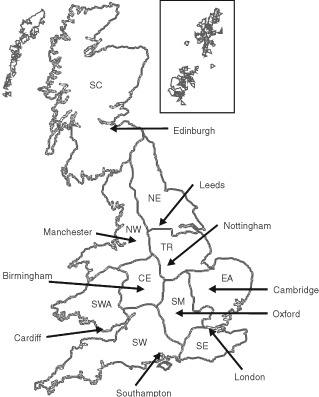
Regional study centres of the UKCCS

The study design and details of case ascertainment and recruitment are given elsewhere (UKCCS Investigators, 2000). The UKCCS covered the whole of England, Scotland and Wales. Ten regional centres (Figure 1

) administered a consistent study protocol with minor regional modifications to satisfy local ethical requirements and practical considerations.

The study aimed to recruit all eligible children aged under 15 years of age diagnosed with malignant diseases during the period 1992–1996 (1991–1994 in Scotland). Two age and sex matched controls were randomly selected from the same (former) Family Health Service Authority (in England and Wales) or Health Board (in Scotland) as the case child (UKCCS Investigators, 2000). The parents/guardians of the children were interviewed, and a wide range of information was collected on the social, occupational and medical histories of the children and their parents. Social class was derived from the occupations of the parents resident with the child at the time of diagnosis, the adult with the highest class taking priority (OPCS, 1990). An alternative measure of socio-economic status was obtained from the 1991 census data by linking postcode of address at time of diagnosis with census enumeration district (or output area in Scotland), and calculating a deprivation score using three variables: proportion of households without a car; proportion of overcrowded households; proportion of persons unemployed (Draper *et al*, 1991; UKCCS Investigators, 2000).

Full details of the methods used for measurement and calculation of mean annual household radon concentrations are described in the main methods paper (UKCCS Investigators, 2000). Briefly, addresses lived in by the child for 6 months or more were targeted for measurement with passive radon detectors, provided by the National Radiological Protection Board (NRPB). Two radon detectors were sent to each address, with instructions to place one in the child's bedroom and one in the main living area. After 6 months a letter was sent recalling the detectors, which were returned to the NRPB for processing and measurement of the cumulative radon exposure. The analyses in this paper only concern the radon results for the address at diagnosis, that is, case children who lived at that address for at least 6 months prior to the date of their diagnosis and the equivalent dates of their controls. Radon levels vary markedly by season and measurements obtained will not precisely reflect the true annual average radon concentration. Seasonal correction factors based on previous studies of ambient household radon were, therefore, applied and weighted average concentrations were calculated, assuming 55% of the child's time was spent in the bedroom and 45% in the living area (Wrixon *et al*, 1988; Darby *et al*, 1998).

Estimates of risk due to radon exposure were obtained from logistic regression using Stata Version 6 (StataCorp, 1999). Radon concentration estimates used were based on measurements of average radon concentration in the home occupied at the time of diagnosis and were seasonally adjusted, using region-specific correction factors. In order to recognise possible dose-response relationships which may not be linear, effects of radon concentrations on childhood cancer risk were investigated for the following pre-defined levels 0–24, 25–49, 50–99, 100–199, and 200+ Bq m^−3^, as used in a UK study of residential radon exposure and lung cancer risk published earlier (Darby *et al*, 1998). Exposure effects were also estimated by dividing radon concentrations into quintiles and deciles.

Because of the known variations in household radon levels, the relationship of radon concentrations with a range of possible measures of socioeconomic status was examined. Increased radon concentration was found to be associated with parents of higher social class, lower deprivation score, being a home owner, and with higher school leaving age (results not shown). Certain aspects of socioeconomic status were potential confounders in our analyses (UKCCS Investigators, 2000). We were also wary of the effects of participation bias, as evidenced by the difference in socioeconomic status between measured and non-measured homes. We therefore included a measure of socioeconomic status in the regression models. The deprivation score was chosen as the prime factor to analyse, rather than social class of parents at diagnosis, because of the large number of unclassifiables in the latter dataset. In addition analyses were conducted on whether the houses that had double glazing or central heating, factors associated with higher household radon concentration, were likely to influence the overall results.

The data were analysed by the major diagnostic divisions of childhood cancer described elsewhere (UKCCS Investigators, 2000), these briefly are Acute Lymphoblastic Leukaemia (ALL), other leukaemias, Non Hodgkin's Lymphoma (NHL), Hodgkin's Disease (HD), Central Nervous System (CNS) tumours and other solid tumours.

## RESULTS

The parents of 3838 children with cancer and 7629 children without cancer were interviewed, representing 87% of eligible cases and 64% of eligible controls (Table 1

**Table 1:**
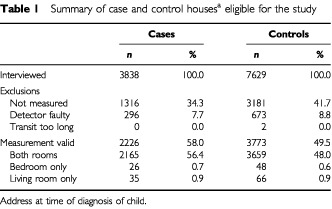
Summary of case and control houses^a^ eligible for the study

). Following interview, radon measurements were obtained from the home at diagnosis of 2226 cases (58% of interviewed cases) and 3773 controls (49% of interviewed controls). Nearly all (97%) estimates were based on readings obtained from both the bedroom and the living room.

The results presented here are largely for unmatched analyses. Matched analyses were also undertaken and are not given in detail in this paper because they add nothing to the results presented, and have wider confidence intervals.

The number of measurements which could be included in an unmatched analysis comparison represents 52% of all UKCCS participants. There were no statistically significant differences between the measured and non-measured homes with regard to the distribution of childhood cancers and their matched controls (Table 2

**Table 2:**
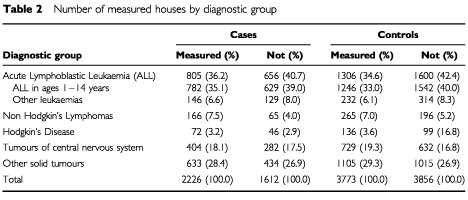
Number of measured houses by diagnostic group

). The characteristics of households where radon measurement were and were not performed are shown in Table 3

**Table 3:**
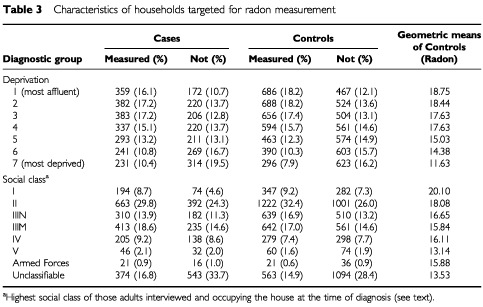
Characteristics of households targeted for radon measurement

. A clear trend with deprivation was evident: the average radon concentration for the most deprived controls being 16.1 Bq m^−3^, compared with the least deprived of 27.2 Bq m^−3^.

The arithmetic mean radon concentration in the homes measured was 24.0 Bq m^−3^, with the mean concentration being slightly lower in case homes than in control homes. Whether homes were distributed by the predefined radon concentrations or by radon quintiles, the proportion of homes at the three higher levels was always greater for controls than cases (Table 4

**Table 4:**
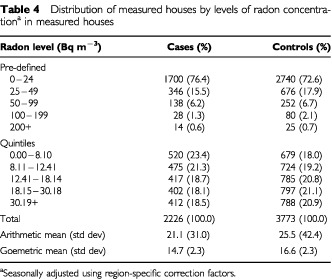
Distribution of measured houses by levels of radon concentration^a^ in measured houses

). Nearly all of children with ALL with the household measure of radon were over 1 year of age at diagnosis (782 out of 805).

Clear negative trends were observed of decreasing childhood cancer risk with increasing radon concentration, most marked in the quintile analysis (Table 5

**Table 5:**
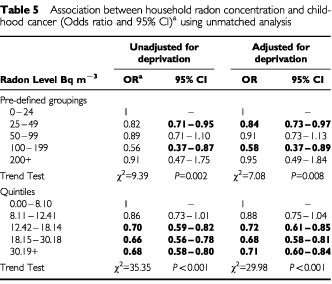
Association between household radon concentration and childhood cancer (Odds ratio and 95% CI) using unmatched analysis

). Adjustment for deprivation made little difference; and similar patterns were evident in each diagnostic group (Table 6

**Table 6:**
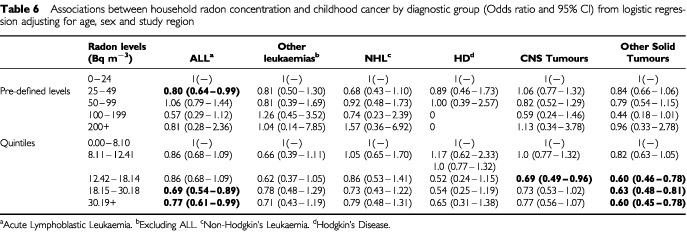
Associations between household radon concentration and childhood cancer by diagnostic group (Odds ratio and 95% CI) from logistic regression adjusting for age, sex, study region and deprivation

). Findings were similar when the analysis was repeated using adjustment for social class (instead of deprivation), and the data were grouped by radon concentration decile, and when radon concentration was treated as a continuous variable.

It is possible that the participation of controls might vary from place to place depending on the local awareness of household radon as a public health problem. However, when control participation rates were correlated with the mean radon concentration by counties (areas of smaller population produce study numbers too low for meaningful analysis) there was no evidence of any such influence (data not shown).

In order to ascertain if an artefact of household measurement was involved, a matched analysis was performed on the basis of predicted radon concentrations for an ‘average’ household within 1 km of the actual case–control house using data from the NRPB. As this analysis was based on national data it was possible to use all household addresses and not confine the analysis to measured households. The results using conditional logistic regression, (with the base-line being <25 Bq m^−3^) give odds ratios for exposures to 25–49 of Bq m^−3^ of 1.1 (0.9–1.2), for 50–99 Bq m^−3^ of 1.1 (0.8–1.6), for 100–199 Bq m^−3^ of 1.0 (0.5–2.1) and for 200+ Bq m^−3^ of 0.6 (0.1–2.9). This suggested that the negative trend might be due to an effect coming from the household measurements themselves. The work of Wrixon *et al* (1988) and Gunby *et al* (1993) suggests that certain household features influence radon concentration. These include flat dwelling (which gives lower average radon concentrations) and double glazing (which gives higher concentrations).

To explore these differences the characteristics of radon concentrations was directly examined (as geometric means) by house type. When control radon concentrations were computed, it was found that house and bungalow measurements were greater than those of flats/maisonettes (17.1 and 9.5 Bq m^−3^ respectively). As this is likely to be due to floor level, an analysis based on the level of the child's bedroom, for example, showed that mean concentrations on the ground floor were 19.7 Bq m^−3^, and above the living room were 16.6 Bq m^−3^, whilst if the bedroom and living room were both above ground level the mean was 9.9 Bq m^−3^. Furthermore the presence of double glazing was associated with increased mean concentration of radon in that household. The mean control level for households with both bedroom and living room having double glazing is 18.1 and without is 14.9 Bq m^−3^. In the case of central heating, control houses without central heating had a mean concentration of 14.2 Bq m^−3^ , with either the bedroom or living room having central heating this became 14.4 Bq m^−3^ but when both were heated the result was 16.3 Bq m^−3^. For each of these housing characteristics the radon concentration was, on average, higher in the homes of controls than cases. However, the addition of these factors to the model made little difference to the overall results already shown. As an example, two aspects of these data for all cancers are shown in Table 7

**Table 7:**
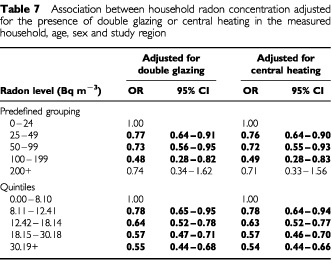
Association between household radon concentration adjusted for the presence of double glazing or central heating in the measured household, age, sex and study region

.

## DISCUSSION

The findings from this large national study offer no support for the suggestion that radon concentration is associated with the incidence of childhood cancer in general, or of leukaemia in particular.

Only 38% of radon measurements were part of a matched set (of a case and at least one control). However, an additional 13% of unmatched radon measurements were available for analysis and the unmatched data are given in Table 4. The analyses of the unmatched findings for the total data clearly show that there is a social class bias in those cases and controls who agreed to participate with more participants in higher socioeconomic groups (based on occupation at the time of diagnosis). Very few households had measured household radon concentration greater than 100 Bq m^−3^ (2.5%). However, the mean radon concentration of 24.0 Bq m^−3^ is very close to the national average and the trends with deprivation, and much of the other results, although statistically significant, are in fact relying on very small concentrations of radon gas.

The results are broadly consistent in the analyses by both quintile and pre-determined levels, with or without adjusting for a deprivation score. Both sets of radon levels show a negative trend but those with the pre-defined levels are less marked. But when 10 equal groupings were created the trend seen in the quintiles remained, with a linear decline in odds ratios from 0.96 (CI 0.74–1.20) in the second lowest radon concentration category to 0.46 (CI 0.34–0.62) in the highest concentration group, with the six highest groups showing statistically significant negative risks.

A similar trend is also seen in all the major diagnostic groups analysed in this paper, although it is not always statistically significant, particularly in the diagnostic categories with smaller case numbers. Such a trend is not consistently seen in the pre-determined level analyses probably due to the small numbers in some of the higher concentration categories. These results are largely unaffected by adjustment for deprivation scores based on census characteristics. In addition the trends are seen in each of the regions participating in this study. Confining the analysis of ALL data to exclude cases aged less than 1 year did not alter the results.

The contribution that the radon (and thoron) gases and their short lived decay products make directly to the bone marrow is modest when contrasted with all the other sources of high - LET exposure to the marrow. For a 1 year-old, inhalation of radon at 20 Bq m^−3^ has been estimated to contribute roughly 10% of the total high LET equivalent dose to the red bone marrow (Simmonds *et al*, 1995 and G Kendall (personal communication)). This would increase to about 20% in a 10-year-old. For a 10-year-old, increasing the concentration of radon breathed by a factor of five would therefore increase the total of high LET equivalent dose by about 1.8 fold, whilst a 10-fold radon increase would increase the high LET total by 2.8 fold. These levels are relatively modest contrasted with other sources of high LET radiation which are predominantly from Po^210^. These variations are unlikely to explain a positive trend, let alone the negative trend seen in this paper. There is also a low-LET contribution to the marrow dose, largely from gamma ray sources, and this is further explored in another paper (see Gamma paper this issue).

When an ecological approach was adopted using NRPB data on localities, the results were generally negative with (excluding the small number in the highest category) a flat distribution. This points to characteristics in the individual households as possibly being important in determining radon concentrations. As it was recognised in this (and other) case–control study that socioeconomic differences occur between cases and controls and between interviewed and first choice controls (UKCCS Investigators, 2000), this area seemed to be likely to hold the possible explanation of the negative trend. Indeed this appears to be the case, when features likely to cause variability of household radon concentrations and census variables are analysed following the work of Wrixon *et al* (1988) and Gunby *et al* (1993). This view is supported by direct studies of case–control differences in double glazing, central heating and dwelling above ground level, which all suggest that controls with a higher ‘socioeconomic status’ (i.e. more likely to have double glazing, central heating and less likely to be a flat dweller) are more likely to be interviewed. Even when controls with low levels of radon concentration occur, the case equivalents are even lower. This could then lead to the negative trend seen across all diagnostic groups.

It is noteworthy that the results for the corresponding UKCCS analysis for gamma radiation in virtually the same households showed no risk but do not show a negative trend (see Gamma paper, this issue). Sources of household gamma radiation are unaffected by whether the dwelling is a flat or by the presence or otherwise of double-glazing and central heating.

It is unlikely that the negative trend is explained by either an adaptive response or a true protective effect. It would be necessary to hypothesise that these explanations held across a very wide range of disease types with known differences in pathogenesis and indeed some, such as HD and NHL have no previously reported associations with ionising irradiation (Mueller, 1996). Most of the scientifically rigorous studies on adaptive responses or protective effects are based on experimental designs delivering tens of milliGrays as stimulating doses and observing responses to larger subsequent challenge doses of radiation to the bone marrow or other tissue (UNSCEAR, 1994). These studies and most others use challenge doses of radiation (usually low-LET) far greater than those seen in the context of this study.

That aside, the overall lack of association in the study is generally consistent either with the more sophisticated of the ecological analyses such as Richardson *et al* (1995) or with the only other large (US) case–control study of Lubin *et al* (1998). Both studies, however, have dealt only with childhood leukaemias. The present study reports upon a larger series of ALL cases than the US study but with far lower numbers in the higher exposed categories. Lubin *et al* (1998) show some signs of lower risks associated with higher levels, but by no means as consistently as in this present study, whilst the US trend was statistically non-significant. This analysis of UKCCS data deals only with concentrations measured after the time of diagnosis in those households occupied at the time of diagnosis and for a minimum period of 6 months prior to that date. One ecological study has suggested a positive relationship between radon concentration at the address at birth and risk of childhood cancer (Gilman and Knox, 1998).

A smaller study from Germany of leukaemias gives internally inconsistent results, showing a significant negative trend in the under 2-year-olds and a positive, but non-statistically significant, trend in the over 2-year-olds for AML (Steinbuch *et al*, 1999). Our study showed, a non-significant but flat relationship in the under 2-year-olds and a negative trend in older cases with AML. It should be noted that in our study childhood cancers other than ALL also show no sign of increased risk associated with radon concentrations but instead a negative trend. A smaller study of leukaemias and certain solid tumours from Germany showed lack of association between leukaemia and radon (Kaletsch *et al*, 1999) whilst a significantly increased risk was observed for CNS tumours, but this was based on only six cases. A recent Swedish ecological analysis has found a risk for higher radon concentration households, but relied on very low numbers in the low concentration group (Kohli *et al*, 2000).

In conclusion, our results are reassuring, in that they do not suggest any detectable public health risk of childhood malignancies in the UK population from radon exposure at the levels measured in the 6 months prior to diagnosis.

### List of investigators

#### Writing Committee

RA Cartwright, G Law, E Roman, KA Gurney, E Gilman, OB Eden, M Mott, K Muir, D Goodhead, G Kendall.

#### Management Committee

KK Cheng, Central Region; N Day, East Anglia Region; RA Cartwright, A Craft, North East Region; JM Birch, OB Eden, North West Region; PA McKinney, Scotland; J Peto, South East Region; V Beral, E Roman, South Midlands Region; P Elwood, South Wales Region; FE Alexander, South West Region; CED Chilvers, Trent Region; R Doll, Epidemiological Studies Unit, University of Oxford; GM Taylor, Immunogenetics Laboratory, University of Manchester, Manchester; M Greaves, Leukaemia Research Fund Centre, Institute of Cancer Research; D Goodhead, Medical Research Council, Radiation and Genome Stability Unit, Harwell; FA Fry, National Radiological Protection Board; G Adams, UK Co-ordinating Committee for Cancer Research.

#### Regional Investigators

KK Cheng, E Gilman, Central Region; N Day, J Skinner, D Williams, East Anglia Region; RA Cartwright, A Craft, North East Region; JM Birch, OB Eden, North West Region; PA McKinney, Scotland; J Deacon, J Peto, South East Region; V Beral, E Roman, South Midlands Region; P Elwood, South Wales Region; FE Alexander, M Mott, South West Region; CED Chilvers, K Muir, Trent Region.

#### Leukaemia Research Fund Data Management Processing Group

RA Cartwright, G Law, J Simpson, E Roman.

A complete list of investigators is given in: The United Kingdom Childhood Cancer Study: objectives, materials, and methods. *Br J Cancer*, 2000 **82:** 1073–1102.
